# Spontaneous social distancing in response to a simulated epidemic: a virtual experiment

**DOI:** 10.1186/s12889-015-2336-7

**Published:** 2015-09-28

**Authors:** Adam Kleczkowski, Savi Maharaj, Susan Rasmussen, Lynn Williams, Nicole Cairns

**Affiliations:** Computing Science and Mathematics, School of Natural Sciences, University of Stirling, Stirling, UK; School of Psychological Sciences and Health; University of Strathclyde, Glasgow, UK; School of Social Sciences, University of the West of Scotland, Paisley, UK

**Keywords:** Epidemics, Social distancing, Agent-based models, Participatory simulation, Virtual experiment

## Abstract

**Background:**

Studies of social distancing during epidemics have found that the strength of the response can have a decisive impact on the outcome. In previous work we developed a model of social distancing driven by individuals’ *risk attitude*, a parameter which determines the extent to which social contacts are reduced in response to a given infection level. We showed by simulation that a strong response, driven by a highly cautious risk attitude, can quickly suppress an epidemic. However, a moderately cautious risk attitude gives weak control and, by prolonging the epidemic without reducing its impact, may yield a worse outcome than doing nothing. In real societies, social distancing may arise spontaneously from individual choices rather than being imposed centrally. There is little data available about this as opportunistic data collection during epidemics is difficult. Our study uses a simulated epidemic in a computer game setting to measure the social distancing response.

**Methods:**

Two hundred thirty participants played a computer game simulating an epidemic on a spatial network. The player controls one individual in a population of 2500 (with others controlled by computer) and decides how many others to contact each day. To mimic real-world trade-offs, the player is motivated to make contact by being rewarded with points, while simultaneously being deterred by the threat of infection. Participants completed a questionnaire regarding psychological measures of health protection motivation. Finally, simulations were used to compare the experimentally-observed response to epidemics with no response.

**Results:**

Participants reduced contacts in response to infection in a manner consistent with our model of social distancing. The experimentally observed response was too weak to halt epidemics quickly, resulting in a somewhat reduced attack rate and a substantially reduced peak attack rate, but longer duration and fewer social contacts, compared to no response. Little correlation was observed between participants’ risk attitudes and the psychological measures.

**Conclusions:**

Our cognitive model of social distancing matches responses to a simulated epidemic. If these responses indicate real world behaviour, spontaneous social distancing can be expected to reduce peak attack rates. However, additional measures are needed if it is important to stop an epidemic quickly.

## Background

Epidemics of infectious disease, such as influenza, are recognized as one of the most serious risks faced by the world today. It is essential to understand how such epidemics develop and how they can be effectively controlled. A variety of studies have shown that human behavioural changes, such as reducing social contacts during outbreaks, can have a significant effect [1–8]. This social distancing response can be particularly useful early in an epidemic, when pharmaceutical interventions such as antiviral drugs and vaccinations might not yet be readily available [4].

Social distancing may be enforced centrally, for example by closing schools and workplaces and cancelling events, or may emerge naturally as a result of individual actions. Our work is aimed at understanding the spontaneous social distancing response. Individual decisions may be driven by many factors, including awareness of infection, advice from governments, and psychological factors. People may also make trade-offs between the social and economic benefits derived from contact and the risk of infection. A number of modelling studies have considered the economic and epidemiological consequences of spontaneous social distancing [5–11]. Caley, Philip, and McCracken [2], for example, found that social distancing behaviour can explain the observation of multiple epidemic waves in the 1918–1919 influenza pandemic. Maharaj and Kleczkowski [5] studied a model which considers individuals’ *risk attitude* as the key parameter driving the response. Simulations of this model showed that the most effective control is achieved when individuals are highly risk-averse, and reduce contacts sharply even at low levels of infection, stopping epidemics quickly. Surprisingly, the worst outcome, as measured in terms of the overall number of cases and the loss of contacts, arises when individuals are moderately risk-averse, reducing their social contacts somewhat, but not sufficiently to suppress the epidemic. This leads to longer epidemics that spread more slowly but eventually still affect the majority of the population. Although this outcome may not be optimal, Lempel et al. [11] show that slowing spread can nevertheless be beneficial by lowering the peak attack rate and reducing stress on healthcare systems.

These studies make clear the importance of understanding social distancing behaviour, but investigating this in the real world is difficult. For obvious reasons, it is not possible to create epidemics for the purpose of research. Most existing studies are based on surveys that either ask people how they would behave during a hypothetical outbreak, or ask them to recall what they did during a recent epidemic [12–14]. Such studies are limited by people’s capacity for imagination or recall. Some studies have looked at data from real epidemics [15–20], for example, public transport records during SARS and swine flu epidemics, but opportunities for collecting such data are limited.

Virtual experiments, using computer simulations as a substitute for reality, can be useful for studying scenarios that cannot practicably be reproduced in the real world or laboratory. Plagues and disease simulations have long been a feature of many popular computer games [21], and have also been used in education [22, 23], but their use for scientific research is relatively recent. Interest was sparked in 2007 by an accidental outbreak that took place in the World of Warcraft online role playing game [24]. A recent study used a computer game to investigate the relationship between self-protective behaviour during epidemics and cost [25]. In this study participants were asked to choose between abstract “high” or “low” cost protective behaviours. Our study also incorporates economic choices, but focusses specifically on social distancing, representing this graphically in a computer game setting. This gives participants a concrete understanding of what kind of behaviour they are being asked to consider and is intended to trigger more realistic responses.

## Methods

We begin with a model of social distancing introduced by Maharaj and Kleczkowski [5], comprising three parts: 1) an epidemic model, representing the dynamics of infection and recovery; 2) a spatial network model, representing relationships amongst individuals which govern the awareness of information about disease and the possibility of physical contact that might spread infection; 3) a cognitive model, representing how an individual responds to the awareness of disease by social distancing behaviour. The model, which was previously used for simulations of social distancing within a homogeneous population [5], is here applied to a population in which individuals may vary in their attitude to risk, both across the population and over time.

Experiments with human participants were carried out to validate the cognitive model by comparing it with the behaviour of real people. Participants played a computer game which incorporated the epidemic and spatial aspects of the social distancing model of [5], but replaced the cognitive aspect with the participant’s own behaviour. Participants also completed a questionnaire based on a psychological theory of health-protective behaviour, the Protection Motivation Theory [26]. The experimental data were used to derive a statistical model of participants’ attitude to risk. This model was then used in a simulation study to explore the outcome in epidemics where people behaved as they did in the experiments.

In the rest of this section we describe in detail the social distancing model (epidemiological, spatial, and cognitive aspects), the population model, the game interface, and the psychological measures.

## Epidemic model

**Fig. 1:**
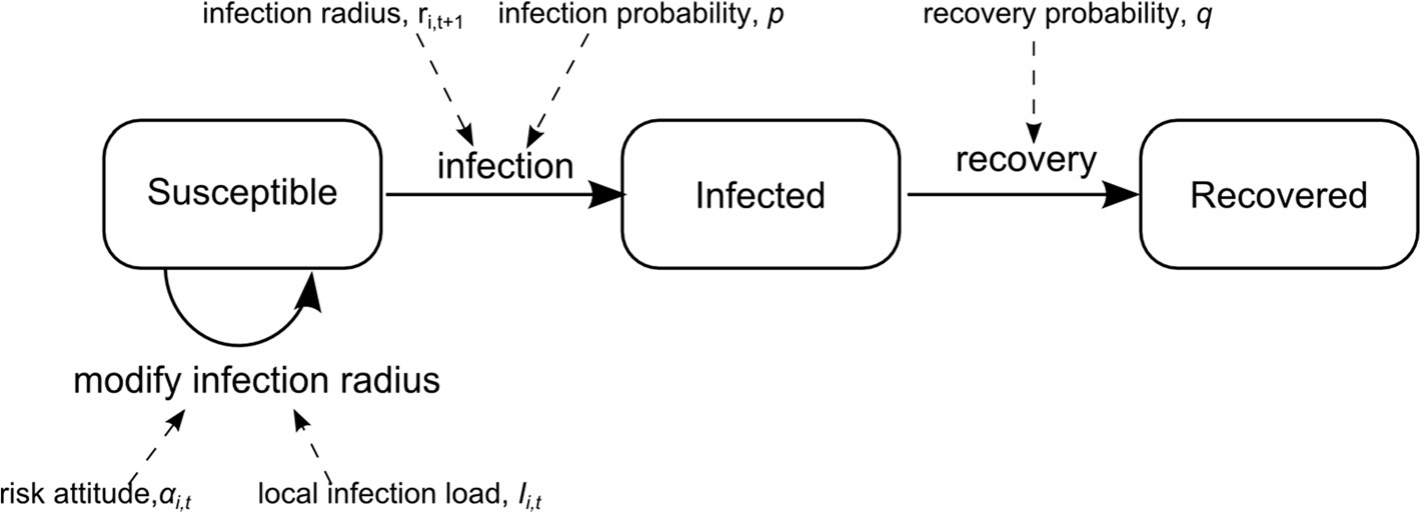
Flow diagram illustrating epidemic spread and social distancing. At each time step *t*, a susceptible individual *i* modifies its infection radius *r*_*i*,*t* + 1_ in response to the local infection load *I*_*i*,*t*_ and current risk attitude *α*_*i*,*t*_. It then makes contact with all neighbours within its infection radius. Each contact with an infected neighbour may cause the susceptible to become infected, with probability *p* = 0.05. At each time step, infected individuals may recover with probability *q* = 0.2, and remain immune from then on

**Fig. 2:**
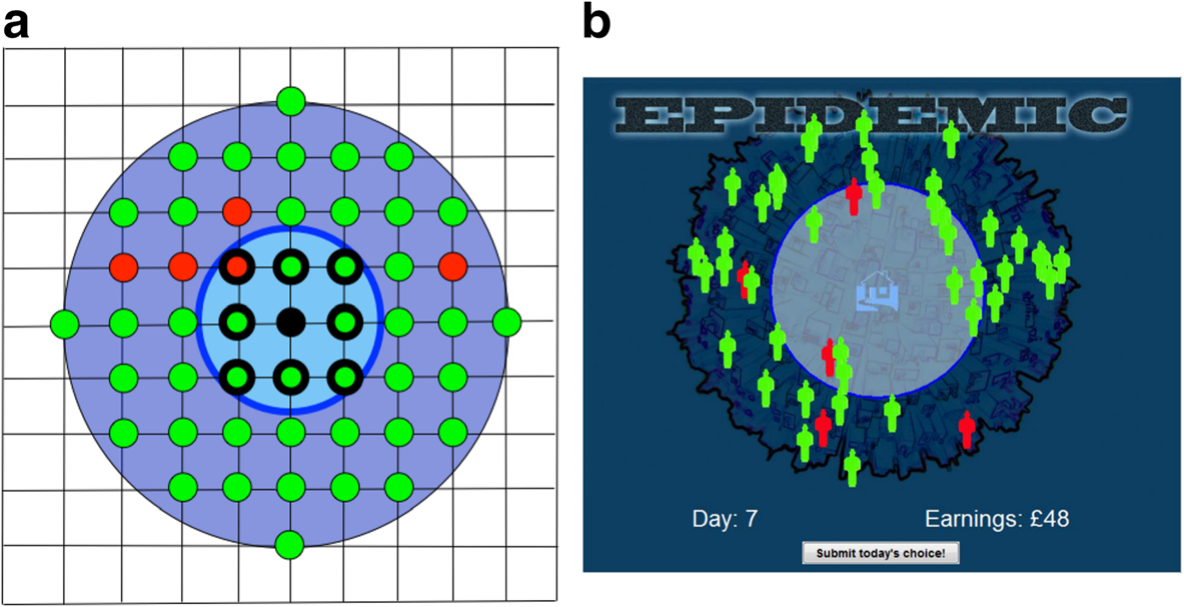
Model network (**a**) compared to the game user interface (**b**). **a** shows a part of the square lattice spatial network with the player-controlled susceptible individual (black circle) in the centre. The player’s awareness neighbourhood (outer, dark blue circle, radius 4) contains infected neighbours (red circles) and non-infected neighbours (green circles). Nodes outside this circle contain individuals that are not visible to the player, but which may transmit infection into the player’s awareness neighbourhood. The inner, light blue circle shows the player’s chosen infection radius, *r*_*i*,*t*_. Here, *r*_*i*,*t*_ = 1.5 and the player will contact 8 neighbours (bold-outlined circles). **b** shows the same scenario as displayed to the player via the game interface. The player controls the size of the infection radius by making the blue circle larger or smaller. Neighbours remain in fixed locations in the underlying lattice, but are displayed in the game interface as animated figures that move around rapidly. This prevents players from wrongly thinking that they can know the exact location of their infected neighbours and attempting to adjust the circle to exclude those locations

Disease transmission and recovery are modelled using the well-known Susceptible-Infected-Recovered (SIR) model [27], adapted to deal with discrete individuals and discrete time. The epidemic model is closely linked with the spatial model, see Figs. 1 and 2. Initially, a fraction *I*_*0*_ of the population are infected and the rest are susceptible. At each time step, each individual makes contact with others; the details of which individuals can contact each other are left to the explanation of the spatial model (below). Each contact between a susceptible and an infected individual may cause the susceptible to become infected, with probability *p*. At each time step, infected individuals may recover, with probability *q*, after which they remain immune.

## Spatial model

The spatial model represents the locations of individuals. It determines what information an individual knows about the extent of the epidemic, and what social contacts the individual can make. The population is assumed to be arranged in fixed locations on a square lattice network, as illustrated partially in Fig. 2a. For each individual *i* on this network, we define a neighbourhood of radius *r* centred on *i* as including all other individuals whose Euclidean distance from *i* is at most *r*. We consider two such neighbourhoods. The *awareness neighbourhood*, which has a fixed radius that is the same for all individuals, represents the information available to the individual: at each time step, *i* is aware of the level of infected cases present in this neighbourhood. The *infection neighbourhood*, whose radius may vary among individuals over time, represents the people with whom *i* has contact, possibly leading to infection transmission. The infection neighbourhood lies within the awareness neighbourhood, and coincides with it when at its maximum size.

## Cognitive model

Social distancing behaviour is represented by having susceptible individuals contract their infection neighbourhood when infection is present locally, thereby reducing the number of social contacts they make. At each time step *t* during the epidemic, each susceptible individual *i* is assumed to be aware of the local infection load *I*_*i,t*_ and may reduce or increase contacts by adjusting the infection radius in the next time step in response to this information. *I*_*i,t*_ is defined as the ratio at time *t* of the number of infected cases within *i*’s awareness neighbourhood to the total number of neighbours within that neighbourhood, and therefore lies in the range [0,1].

**Fig. 3:**
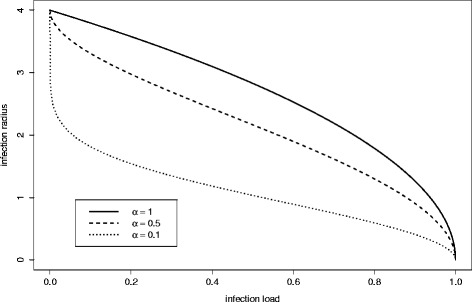
Cognitive model of social distancing. Equation 1 is illustrated for three values of *α*_*i*,*t*_. The plots show the reduction in infection radius in response to infection load. Lower values of *α*_*i*,*t*_ represent more cautious risk attitudes, and cause a sharper reduction in infection radius for a given infection load

In previous work [5] we proposed a cognitive model, Eq. 1 and Fig. 3, to represent this behaviour. Equation 1 describes how the individual adjusts the infection radius in the next time step, *r*_*i,t+1*_, in response to the current local infection load, *I*_*i,t*_. The parameter *α*_*i*,*t*_ represents the individual’s attitude to risk, and is a positive real number, unbounded from above. Lower values of *α*_*i*,*t*_ represent more cautious, or risk-averse attitudes, and produce a larger reduction in the infection radius for a given infection load. Equation 1 has the property that when no infection is present individuals will choose the maximum possible infection radius, *r*_*0*_*.*

1 $$ {r}_{i,t+1}={r}_0\sqrt{1-{\left({I}_{i,t}\right)}^{\alpha_{i,t}}} $$

[Note: The cognitive model in [5] described reducing the *number* of contacts rather than the infection radius. In this paper we use the same model but focus on the infection radius for ease of comparison with the computer game. The square root in Eq. 1 comes from the fact that this radius is proportional to the square root of the number of contacts].

## Population model

In our previous simulation study [5] it was assumed that all individuals within the population have the same risk attitude, and that this remains fixed over time. Here we relax this assumption and introduce the possibility of heterogeneous and time-varying risk attitudes. We propose that an individual’s risk attitude at a given time step is a combination of up to four factors: the baseline risk attitude in the population, the variation due to the individual, the individual’s variation over repeated games, and the individual’s variation over time within a game. A statistical model of this variability will be derived from experimental data, as described later.

## Computer game

A computer-game tool was created to allow experimental investigation of social distancing behaviour in real people. The game is based upon an underlying agent-based simulation of an epidemic. It is played by a single player who controls the behaviour of one agent (representing a susceptible individual), interacting with a population of computer-controlled agents. This technique is known as *agent-based participatory simulation* [28–30].

The simulation uses the epidemic model described above, with parameters *p =* 0.05 and *q* = 0.2. Initially, 6 % of the population are infected, and the rest are susceptible. These values were chosen as they generate simulated epidemics that expose participants to a wide range of infection levels, allowing risk attitudes to be estimated. The simulation also uses the spatial model described above, with 2500 individuals arranged on a 50 × 50 square lattice, and with the awareness radius (and maximum infection radius) *r*_*0*_ set at 4. This means that each individual may contact at most 48 neighbours at each time step, Fig. 2a. Note that the use of a square lattice network makes it necessary to use epidemic parameters that are much higher than those used in mean-field or random network models. This is because the network structure exhibits spatial correlations that suppress transmission [31] and lower the effective reproductive ratio.

Social distancing behaviour in the game is determined by the player, who is given information about the current local infection load *I*_*i*,*t*_ and is allowed to adjust *r*_*i*,*t* + 1_ freely. Equation 1 is inverted to calculate the player’s effective risk attitude, *α*_*i*,*t*_, so that:

2 $$ {\alpha}_{i,t}=\frac{ \log \left(1-{\left(\frac{r_{i,t+1}}{4}\right)}^2\right)}{ \log \left({I}_{i,t}\right)} $$

This inverse is defined when infected cases are present and the player reduces contact (*I*_*i*,*t*_ > 0, *r*_*i*,*t* + 1_ < 4). In all other cases, the player’s risk attitude is not calculated.

**Fig. 4:**
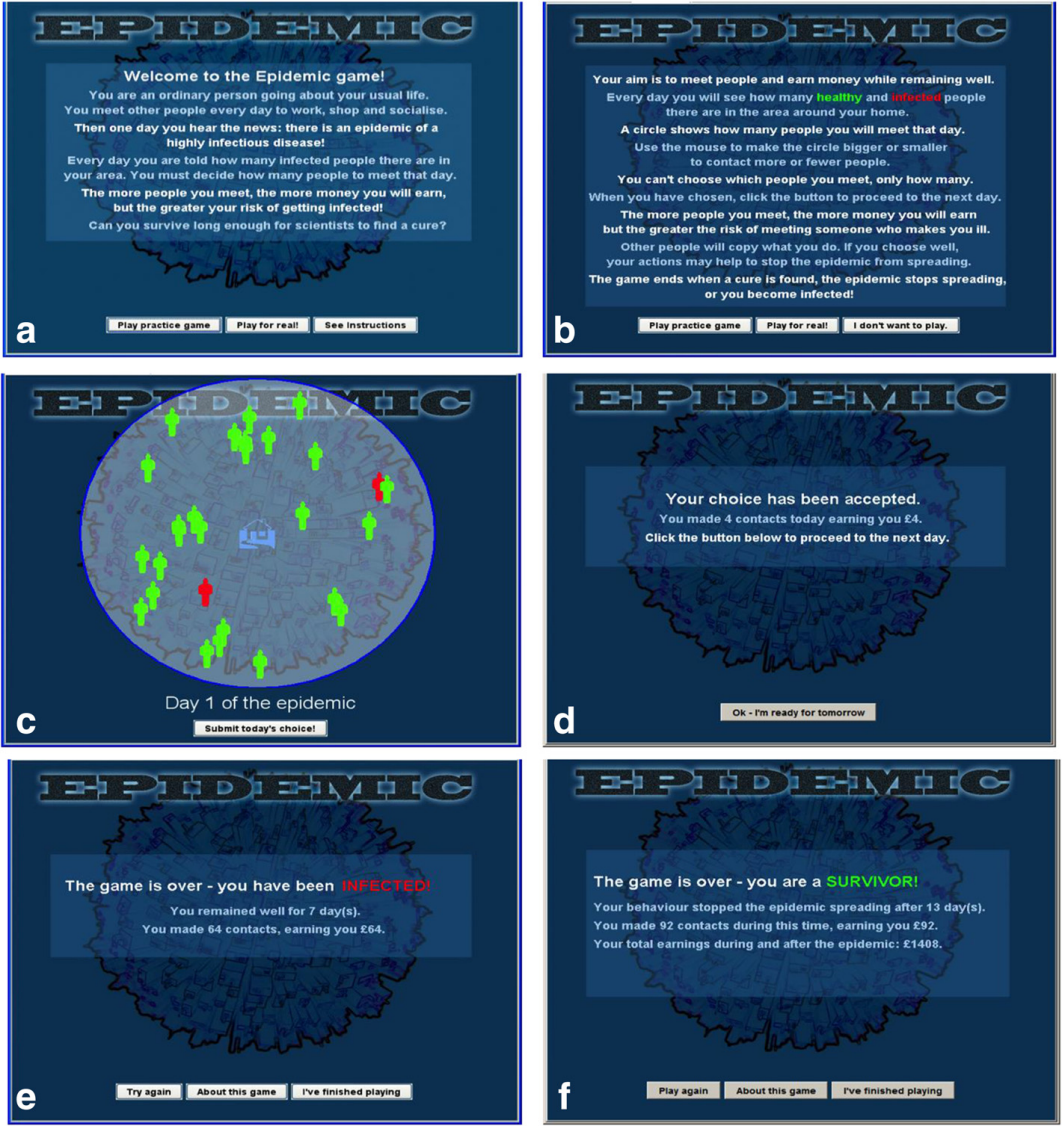
Game interface. **a** Welcome screen. **b** Instructions. **c** Play screen showing infection load on the current day. The player chooses the number of contacts to make by adjusting the circle and then clicks the submit button. **d** Outcome if the player remains healthy; points are given for contacts made and game play continues. **e** Outcome if the player becomes infected; game play ends. **f** Outcome if the player remains well until all infected cases have recovered; game play ends and the player receives extra points. Play may also end due to time-out after 60 days (not shown)

The player interacts with the underlying simulation via a computer game interface (Fig. 2b and Fig. 4). After viewing instructions, Fig. 4a-b, the player sees a visualization of the local neighbourhood, Fig. 4c, with animated red and green figures representing, respectively, infected and non-infected neighbours. The player uses the mouse to adjust the size of a circle, Fig. 2b, representing the chosen infection radius *r*_*i*,*t* + 1_, and then clicks a button to submit the choice for the next day. The player’s effective risk attitude *α*_*i*,*t*_ is then calculated as described above, and is applied to adjust the contacts made by the computer-controlled agents. [It is necessary for the computer-controlled agents to modify their contacts; if they do not do so, the simulated epidemic rapidly becomes invasive and the game ends before sufficient data can be collected. By having the computer-controlled agents adopt the same risk attitude as the player, the game maintains consistency with the model used in previous work [5], where all members of the population have the same risk attitude, but not necessarily the same infection radius due to differing local infection loads].

One step of the epidemic dynamics is then carried out, causing individuals to either remain in the same state or to change from susceptible to infected or from infected to recovered. If the player becomes infected, the game ends and the message “You have been infected” is displayed, Fig. 4e. If the player manages to remain uninfected until all infected cases recover, the game ends, and the message “You are a survivor” is displayed, Fig. 4f. To prevent excessively long play, the game also ends after a timeout period of 60 days. If the game does not end, the player is credited with a number of “points” corresponding to the number of contacts made during the current day, and play continues to the following day, Fig. 4d. Players who survive an epidemic lasting less than 60 days are credited with additional points equivalent to making full contacts during the period from the end of the epidemic to the 60-day maximum. This removes any incentive for players to deliberately attempt to prolong the epidemic so as to amass more points.

## Psychological measures

Protection Motivation Theory (PMT) is a psychological framework for understanding what motivates people to change their behaviour in order to protect their health [26]. According to PMT, people’s motivation to protect themselves from health threats is determined by four main factors: perceived *severity* of the threat, perceived *vulnerability* to the threat, perceived efficacy of the recommended protective behaviour (*response-efficacy*), and belief in one’s ability to carry out this behaviour (*self-efficacy*). Support for PMT measures as predictors of behaviour during epidemics comes from studies of responses to recent outbreaks of SARS, avian flu and swine flu [32–35].

After playing the game, participants in our study completed a self-report questionnaire which asked them to state the extent to which they agreed or disagreed with several statements designed to measure their beliefs according to PMT: perceived severity, perceived vulnerability, fear, response efficacy, self-efficacy, response cost, and intention. Table 1 shows examples of these statements. The questionnaire was based upon one used previously in a study of exercise participation [36].

**Table 1:** Example statements for measuring PMT beliefs

Perceived severity of illness:	
• If I were to develop an infectious disease (e.g. flu) I would suffer a lot of unpleasant symptoms.	
• Developing an infectious disease would be unlikely to cause me to die prematurely.	
Perceived vulnerability:	
• My chances of developing an infectious disease (e.g. flu) in the future are likely.	
• I am unlikely to develop an infectious disease (e.g. flu) in the future.	
Response efficacy:	
• If I were to engage in social distancing (e.g. by avoiding public transport and social events) I would lessen my chance of developing an infectious disease.	
Self efficacy:	
• I am discouraged from engaging in social distancing during times of infectious disease, because I feel it would be difficult to do so.	
• I feel confident in my ability to engage in social distancing during times of infectious disease.	

## Experiments

The experiments involved 230 participants recruited at two locations: 200 individuals were visitors to the Glasgow Science Centre, a large science museum that is popular with families, and 30 were students at a university in the west of Scotland. There were 109 male and 121 female participants, with ages ranging from 18 to 89 years and a mean age of 32.4 years, standard deviation 14.22. The age distribution shows a large peak around 20, representing students, and another around 45, most likely representing parents visiting the Glasgow Science Centre. Participants first played repeated rounds of the computer game and then completed the questionnaire. There was no payment or other reward given for taking part in the experiment.

## Ethics statement

This study was approved by the School of Psychological Sciences and Health Ethics Committee at the University of Strathclyde. Informed written consent was obtained from all participants before commencing the study. For descriptive purposes, all participants were asked to indicate their age and gender (male/female). No other confounding variables were included.

## Results

Each of the 230 participants played between 1 and 9 games, yielding 852 games to be analysed. The number of time steps in each game ranged from one to the maximum permissible length of 60. The first game was designated as a practice game for getting familiar with the game interface, and results indicate that it was indeed used for this purpose. The mean duration of first games was 7.8 steps as compared to 14.7 steps for subsequent games (*t*-test, p < 0.001, 565df), whereas there was no difference in duration between second and third games (*t*-test, p = 0.74, 416df), and the same applied to subsequent ones. Only three participants played more than 5 games. We therefore considered only games 2 to 5 in the analysis. We also removed all games for which the duration was just one step (5.7 %), leaving 589 games.

## Comparison of social distancing model with responses in experiments

**Fig. 5:**
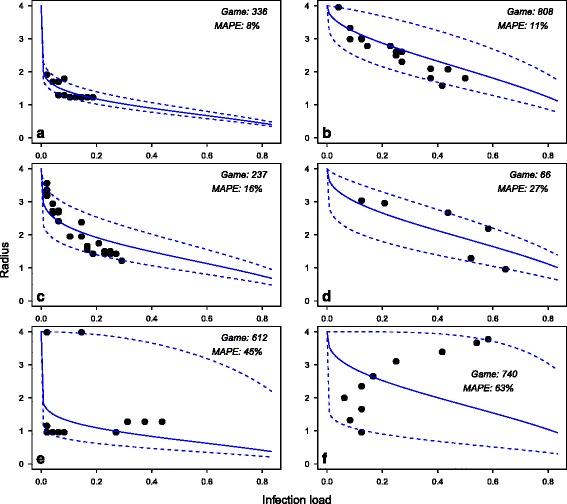
Best and worst examples of model fit. The player’s chosen infection radius *r*_*i*,*t* + 1_ is plotted against the corresponding infection load, *I*_*i*,*t*_, at all time steps *t* for six games. (A “game” refers to a single round of the computer game, played by a distinct participant). The solid line shows the prediction based on Eq. (1) with *α* calculated as a geometric mean of the values of *α*_*i*,*t*_ at each step in the given game; broken lines are based upon the 95 % confidence intervals for *α*. Games number 336, 808, 237 and 66 (panels (**a**)to (**d**), respectively) are examples of a very good fit (low MAPE scores), whereas games 619 (panel (**e**)) and 749 (panel (**f**)) are examples where the fit was bad

In our cognitive model of social distancing (Eq. 1), an individual *i* responds to the infection pressure, *I*_*i*,*t*_, by adjusting the infection radius, *r*_*i*,*t* + 1_. This response was observed in experiments but was characterised by large variability; for examples see Fig. 5. Using Eq. 2, we calculated the participant’s risk attitude *α*_*i*,*t*_ at each time step in all games played, ignoring time steps where this could not be calculated. The distribution of *α*_*i*,*t*_ was found to be highly skewed (mean 0.20, standard deviation 0.35, skewness 8.8). A log transformation [37] was applied to reduce skewness, as the distribution of log *α*_*i*,*t*_ is approximately normal (mean -2.2, standard deviation 1.0, skewness 0.4). We therefore refer to log *α*_*i*,*t*_ (or the log risk attitude) in the analysis that follows.

The model predicts a gradual decrease in *r*_*i*,*t* + 1_ in response to increased infection pressure, *I*_*i*,*t*_, Fig. 3. For each game that was played, we take the experimentally observed value of log *α*_*i*,*t*_, averaged over all time steps in the game, to find the value of ${\widehat{r}}_{i,t+1}$ predicted by Eq. 1, for every experimentally observed value of *I*_*i*,*t*_. This allows us to compare the predictions of Eq. 1 with the actual response of the participant who played that game. The results range from a very good agreement with the model (Fig. 5a-d), through indifference (Fig. 5e), to increase in *r*_*i*,*t* + 1_ (Fig. 5f). We quantify the quality of the model predictions by using the Mean Absolute Percentage Error averaged over each game *k*, $MAP{E}_k=100/{n}_k\times {\displaystyle \sum_t\left|{\displaystyle {r}_{i,t+1}}-{\displaystyle {\widehat{r}}_{i,t+1}}\right|/{\displaystyle {r}_{i,t+1}}}$ (where |*x*| denotes the absolute value of *x* and *n*_*k*_ is the number of steps in game *k*), [38]. This procedure yields a distribution of *MAPE*_*k*_ for all games. The mean value of *MAPE*_*k*_ is 14 %, but the distribution is strongly influenced by a small number of games where the discrepancy between the data and the model is particularly severe (cf. games 612 and 740 in Fig. 5e and f). 57 % of the games have *MAPE*_*k*_ smaller than 10 %; this number increases to 88 % with *MAPE*_*k*_ smaller than 25 %. Thus, for the majority of the games, Eq. 1 captures the behaviour of the participants.

## Statistical modelling of participant behaviour

The experimentally-observed log risk attitude varies among players. For each player, it varies among games, *k* = 2,…,5, and within a single game it varies over time. We explore the data through a series of linear models with the aim of deriving a model of population heterogeneity and time variation in risk attitudes. The models, labelled A-D, are presented in Table 2.

**Table 2:** Models of (log) risk attitude as a function of games, participants and time

Model	Formula	Population *μ*	Participants *λ*_*i*_	Games *η*_*i*,*k*_	Time *ε*_*i*,*k*,*t*_	AIC
A	log(*α*_*i*,*k*,*t*_) = *μ* + *λ*_*i*_ + *η*_*i*,*k*_ + *ε*_*i*,*k*,*t*_	–1.934	0.7354	0.2730	0.5833	14502
B	log(*α*_*i*,*k*,*t*_) = *μ* + *λ*_*i*_ + *ε*_*i*,*k*,*t*_	–2.004	0.7935		0.6719	15835
C	log(*α*_*i*,*k*,*t*_) = *μ* + *η*_*k*_ + *ε*_*i*,*k*,*t*_	–2.382		0.1697	0.9544	20235
D	log(*α*_*i*,*k*,*t*_) = *μ* + *ε*_*i*,*k*,*t*_	–2.325			0.9628	20348

Random effects (*λ*_*i*_, *η*_*i*,*k*_) and time variability (*ε*_*i*,*k*,*t*_) are distributed according to a normal distribution with average 0 and standard deviation listed in the table. AIC is the Akaike Information Criterion [40]

Model A takes into account all factors. The first term, *μ*, represents the average log risk attitude of the population. The second term, *λ*_*i*_ describes the variability among participants, so that *μ* + *λ*_*i*_ represents the log risk attitude for participant *i* averaged over their games and time steps. The third term, *η*_*i*,*k*_ describes the differences between different games played by each participant, so that *μ* + *λ*_*i*_ + *η*_*i*,*k*_ represents the log risk attitude adopted by participant *i* in game *k*. Finally, the last term, *ε*_*i*,*k*,*t*_, represents the variation in the log risk attitude over time steps within a single game.

Model A is a linear model with nested effects. The participant effect is clearly a random effect, as we are interested in the variation for the whole population from which the participants form a random sample. Although in our design we limit the number of games, we are interested in how people would respond in an unspecified number of games; this allows us to treat the game effect as random as well. We treat the last term as an error term, as it represents the random variation in the response from step to step.

We also study three reduced models: Model B, where only the participant effect is considered; Model C, where only the game effect is considered; and Model D, where the log risk attitude is assumed to be constant across participants and games. Results of the models with quality of fit based on the Akaike Information Criterion (AIC) are given in Table 2.

**Fig. 6:**
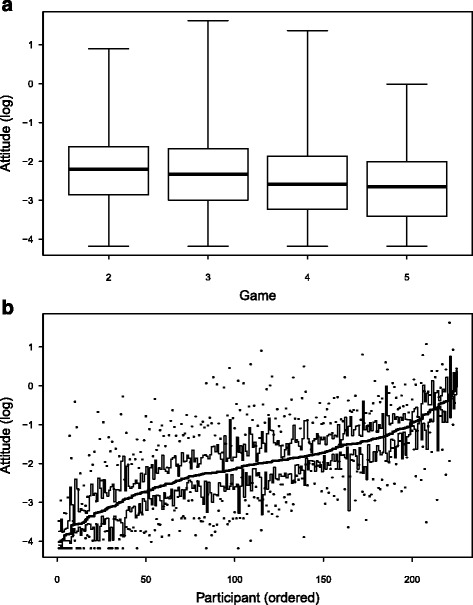
Distribution of (log) risk attitudes across (**a**) games and (**b**) participants. In (**b**), the 230 participants are ordered according to median log risk attitude, with the most cautious at the left. The figure shows box plots of the log risk attitude for each participant. The solid central line shows the median for each participant, surrounded by quartiles (thinner black line) with ranges shown as dots

The effect of the game appears to be small, Model C, Fig. 6a. There appears to be some trend towards lower log risk attitude and hence towards more cautious behaviour as more games are played, but it is very weak (when the game effect is added, the AIC drops from 20348 (Model D) to 20325).

There is, however, a strong participant effect, Model B, Fig. 6b. Although the variation within each participant (due to the combined effect of game and time) is considerable, the AIC shows a significant decrease when Model B is compared with Model D (cf. AIC = 15835 for Model B with 20348 for Model D), or Model C.

Finally, the most complex model, Model A, improves the AIC from 15835 for Model B to 14502. This decrease is significant, so Model A is used for the simulations described in the next section.

## Consequences of experimentally-observed behaviour

To explore the consequences of the experimentally-observed behaviour in an epidemic, we performed simulation studies. The simulation model used is the same agent-based model underlying the computer game, with the difference that all individuals are computer-controlled and carry out social distancing behaviour according to Eq. 1.

We looked at the effect of sweeping through the range of log risk attitudes seen in the experiment. Simulated agents were programmed to behave as described in Model A, with variation amongst participants, games, and over time steps as in Table 2, but with the population baseline log risk attitude (μ) systematically swept across the range [−5,1]. This range includes all the log risk attitudes observed in the experimental participants. The epidemic and spatial models were parameterized as in the experiments, that is, with *p* = 0.05, *q* = 0.2, and the awareness radius and maximum infection radius both equal to 4. Each simulated epidemic was allowed to run until it was complete, that is, there were no infected individuals left in the population, or for a maximum of 1000 time steps. Each run was replicated 100 times. To provide a baseline for comparison, we also performed simulations using the same epidemic and spatial parameters, but without the social distancing response.

**Fig. 7:**
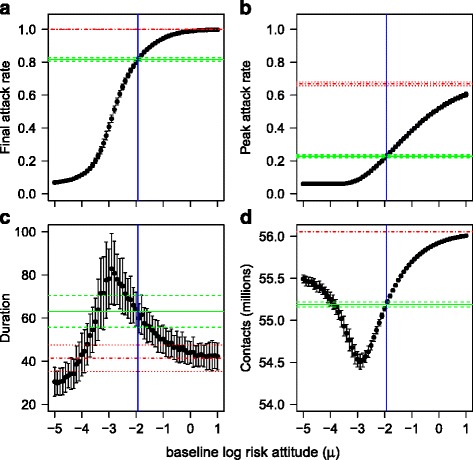
Experimental behaviour and simulated outcomes. The results of simulations with a population behaving according to Model A, and with epidemic and spatial parameters as in the experiments, but with varying baseline log risk attitude (μ), is shown as black points with error bars representing ± one standard deviation based on 100 replicates. Solid (*blue*) vertical lines show the experimentally observed *μ* = − 1.934 as in Model A. Solid (green) horizontal lines show the outcomes from 100 simulations with the experimentally observed μ, with dashed horizontal lines corresponding to ± one standard deviation. Broken (red) horizontal lines show outcomes from 100 simulations with no social distancing response, with dotted horizontal lines corresponding to ± one standard deviation. **a** shows the attack rate, that is, the proportion of recovered individuals when the epidemic is over. **b** shows the peak attack rate. **c** shows the duration. **d** shows the amount of social contact over 1000 time steps. It is assumed that all individuals resume full social contact once the epidemic is over

Figure 7 shows the results of the simulation study. When μ is low, the behaviour is very cautious and even a small infection pressure results in a strong contraction of the infection radius. This stops the disease spread, resulting in very limited epidemics of short duration (left hand side of Fig. 7a and c). As a result a high overall level of social contact is achieved, due to the resumption of full contact once the epidemic is over (left-hand side of Fig. 7d). The peak attack rate is also very low (left hand side of Fig. 7b).

At the other extreme, high values of μ result in behaviour that does not react to even high infection load, leading to short epidemics that quickly reach most if not all of the population (right hand side of Fig. 7a and c). Again, the overall number of contacts is relatively high, due to the short epidemic duration (right hand side of Fig. 7d). The peak attack rate is also high (right hand side of Fig. 7b).

A sharp transition occurs at middle values of μ, separating the cautious behaviour that can suppress spread and the more relaxed behaviour that leads to a major outbreak. Interestingly, most participants displayed a log risk attitude that was either near this transition or towards the more relaxed strategy, cf. Figs. 6 and 7 (vertical blue line). The outcomes of this behaviour (Fig. 7, horizontal solid green line) can be contrasted with the outcomes when there is no response (Fig. 7, horizontal broken red line). The experimentally-observed response results in epidemics that are invasive, but with an attack rate that is lower than the outcome with no response (18 % reduction, 0.82 compared to 1.00; 1.00 corresponding to the whole population infected). However, there is a substantial reduction in the peak attack rate (44 %; 0.23 compared to 0.67 without response). The average epidemic duration is increased (63 time steps compared to 41) and the total number of contacts taking place is reduced (55.19 million, compared to 56.05 million, counted over the maximum run length of 1000 steps, with the assumption that full contact is resumed once the epidemic is over).

Figure 7 also shows that small shifts from the experimentally-observed response towards more cautious behaviour (smaller μ) reduce the attack rate and peak attack rate further, but also prolong the epidemic and reduce the number of contacts. In order to improve all four outcomes, it is necessary to substantially reduce μ (to around −4, corresponding to an average risk attitude of 0.02).

**Fig. 8:**
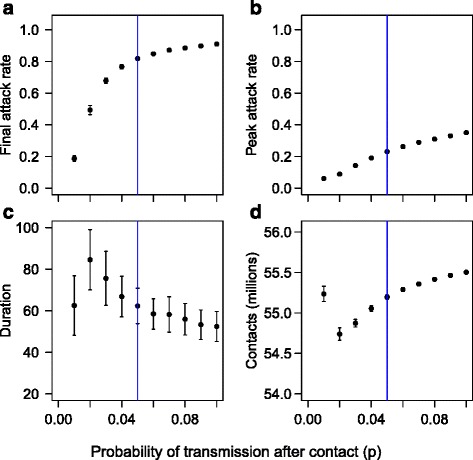
Local sensitivity of simulation outcomes to probability of transmission. The results of simulations with a population behaving according to Model A, with epidemic and spatial parameters as in the experiments, except that *p* is varied in a narrow range [0, 0.1] around the experimental value (*p* = 0.05, indicated by a vertical line in the figures). Each black point represents the mean of 100 replicates, with error bars representing ± one standard deviation. **a** shows the attack rate. **b** shows the peak attack rate. **c** shows the duration. **d** shows the overall level of social contact during 1000 time steps. It is assumed that all individuals resume full social contact once the epidemic is over

**Fig. 9:**
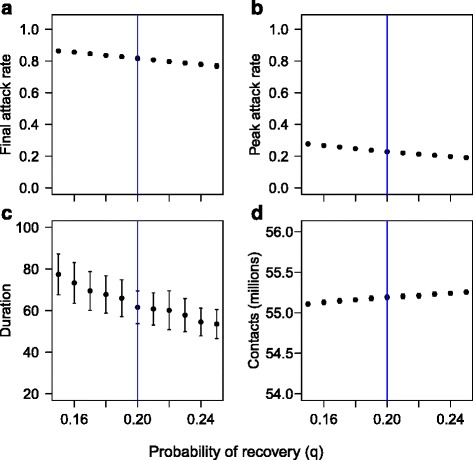
Local sensitivity of outcomes to probability of recovery. The results of simulations with a population behaving according to Model A, with epidemic and spatial parameters as in the experiments, except that *q* is varied in a narrow range [015,0.25] around the experimental value (*q* = 0.20, indicated by a vertical line in the figures). Each black point represents the mean of 100 replicates, with error bars representing ± one standard deviation. **a** shows the attack rate. **b** shows the peak attack rate. **c** shows the duration. **d** shows the overall level of social contact during 1000 time steps. It is assumed that all individuals resume full social contact once the epidemic is over

Local sensitivity analysis was performed to explore the effect of slightly varying the values of *p* and *q* used in the simulations. The results are shown in Figs. 8 and 9. Small increases in *p* lead to increases in the attack rate, peak attack rate, and number of contacts, and a reduction in the duration. Small increases in *q* lead to reductions in the attack rate, peak attack rate and duration, and an increase in the number of contacts.

## Comparison with psychological measures

Data from questionnaires were used to measure the four factors of Protection Motivation Theory, namely, perceived severity of illness, perceived vulnerability, response-efficacy, and self-efficacy. A detailed analysis of the questionnaire results has been published separately [39]. It was found that fear, response efficacy, and self-efficacy were all significant predictors of intention to engage in social distancing behaviour. However none of the PMT variables were significant predictors of social distancing behaviour during the computer game task itself. Further work is needed to investigate the reasons for this. Some possible explanations are discussed in [28] and [39].

## Discussion and conclusions

### Evidence found for social distancing

Our study found evidence of social distancing behaviour within a virtual experiment setup. Participants showed large variability in their behaviour, but in general they did practice social distancing by reducing their social contacts in response to local infection pressure. There was good agreement between participants’ responses and our cognitive model of social distancing behaviour (Eq. 1).

### Effectiveness of spontaneous social distancing for controlling epidemics

A key conclusion of our study is that the spontaneous social distancing response appears to be too weak to be relied upon as a sole means of controlling epidemics. Our study shows that in the experimental scenario, with a population behaving similarly to the experimental participants, epidemics are invasive and would affect over 80 % of the population. If this is indicative of real world behaviour, it suggests that policy makers may not be able to rely exclusively on spontaneous social distancing to control epidemics but should supplement this with other measures such as enforced social distancing (e.g., school closures, event cancellations) and similar strategies. Our cognitive model assumes that individuals have perfect knowledge of the infection load within the awareness radius. Our previous study [5] considered the situation where the infection load is over- or under-estimated (by using an awareness radius that is, respectively, larger or smaller than the maximum infection radius). In both cases, the effectiveness of social distancing is reduced.

A second key observation is that spontaneous social distancing leads to a substantial reduction in the peak attack rate. In a real world situation, this could be very beneficial for preventing healthcare systems from being overwhelmed [11]. However, this benefit must be balanced against other less desirable outcomes, such as the prolongation of the epidemic and the reduction in social contacts.
